# Supplementary material for: GelMap: intrinsic calibration and deformation mapping for expansion microscopy
Source: Nat Methods. 2023 Sep 18;20(10):1573–80. doi: 10.1038/s41592-023-02001-y (PMC10555834; doi:10.1038/s41592-023-02001-y)
Supplement: Supplementary file 1 — Reporting Summary [file 41592_2023_2001_MOESM1_ESM.pdf]

nature portfolio

Corresponding author(s): Lukas Kapitein

Last updated by author(s): Jul 28, 2023

Reporting Summary

Nature Portfolio wishes to improve the reproducibility of the work that we publish. This form provides structure for consistency and transparency in reporting. For further information on Nature Portfolio policies, see our Editorial Policies and the Editorial Policy Checklist.

Statistics

For all statistical analyses, confirm that the following items are present in the figure legend, table legend, main text, or Methods section.

n/a	Confirmed	
☐	☒	The exact sample size (*n*) for each experimental group/condition, given as a discrete number and unit of measurement
☐	☒	A statement on whether measurements were taken from distinct samples or whether the same sample was measured repeatedly
☐	☒	The statistical test(s) used AND whether they are one- or two-sided<br>*Only common tests should be described solely by name; describe more complex techniques in the Methods section.*
☒	☐	A description of all covariates tested
☒	☐	A description of any assumptions or corrections, such as tests of normality and adjustment for multiple comparisons
☐	☒	A full description of the statistical parameters including central tendency (e.g. means) or other basic estimates (e.g. regression coefficient) AND variation (e.g. standard deviation) or associated estimates of uncertainty (e.g. confidence intervals)
☐	☒	For null hypothesis testing, the test statistic (e.g. *F*, *t*, *r*) with confidence intervals, effect sizes, degrees of freedom and *P* value noted<br>*Give P values as exact values whenever suitable.*
☒	☐	For Bayesian analysis, information on the choice of priors and Markov chain Monte Carlo settings
☒	☐	For hierarchical and complex designs, identification of the appropriate level for tests and full reporting of outcomes
☒	☐	Estimates of effect sizes (e.g. Cohen's *d*, Pearson's *r*), indicating how they were calculated

Our web collection on statistics for biologists contains articles on many of the points above.

Software and code

Policy information about availability of computer code

Data collection	No software was used for data collection
Data analysis	All imaging processing was done using FIJI v1.54e (Schindelin et al., 2012). Deformation correction was performed using publicly available code. The code/workflow for measuring absolute deformation and correcting is available in (Jurriens et al. 2020; doi: 10.1016/bs.mcb.2020.04.018). The code for quantifying measurement error between pairs of points for a measurement length is described in (Damstra et al. 2022; doi: 10.7554/eLife.73775), originally from (Chen et al. 2015; doi: 10.1126/science.1260088). All data visualization and statistics were performed using GraphPad Prism 9.

For manuscripts utilizing custom algorithms or software that are central to the research but not yet described in published literature, software must be made available to editors and reviewers. We strongly encourage code deposition in a community repository (e.g. GitHub). See the Nature Portfolio guidelines for submitting code & software for further information.

Data

Policy information about availability of data

All manuscripts must include a data availability statement. This statement should provide the following information, where applicable:

- Accession codes, unique identifiers, or web links for publicly available datasets
- A description of any restrictions on data availability
- For clinical datasets or third party data, please ensure that the statement adheres to our policy

The datasets generated and/or analyzed during the current study are available on figshare (https://doi.org/10.6084/m9.figshare.21923418). The design files used to generate photomasks for the current study are available upon request, as they require an MTA.

Human research participants

Policy information about studies involving human research participants and Sex and Gender in Research.

Reporting on sex and gender	Our study did not involve human research participants
Population characteristics	Our study did not involve human research participants
Recruitment	Our study did not involve human research participants
Ethics oversight	Our study did not involve human research participants

Note that full information on the approval of the study protocol must also be provided in the manuscript.

Field-specific reporting

Please select the one below that is the best fit for your research. If you are not sure, read the appropriate sections before making your selection.

☒ Life sciences ☐ Behavioural & social sciences ☐ Ecological, evolutionary & environmental sciences

For a reference copy of the document with all sections, see nature.com/documents/nr-reporting-summary-flat.pdf

Life sciences study design

All studies must disclose on these points even when the disclosure is negative.

Sample size	To measure expansion gel anisotropy, three regions from three separate gels were analysed. For each replicate (expansion gel FOV), the entire FOV was analysed and all data points included in analysis of expansion factor and squareness. To assess the impact of GelMap on cell morphology, two independent experiments were performed and more than 50 cells per replicate analysed. To assess the impact of GelMap on expansion factor, three independent experiments were performed. For quantification of Bassoon/Homer1 separation, 3 biological replicates were used and a total of more than 50 synapses analysed. The number of replicates for each experiment was determined by ensuring that a large sample size was obtained.
Data exclusions	No data points were excluded from analysis.
Replication	Reproducibility of correction of deformation using GelMap is demonstrated by a full replication of Fig. 2C-G, in Fig. S6F-I.
Randomization	There are no distinct experimental groups in this study.
Blinding	For Fig. S6A (measurement of macroscopic expansion factor by unbiased participants), participants were not given any indication in advance of the 'true' expansion factor. Blinding was not required for other experiments as there was no group allocation.

Reporting for specific materials, systems and methods

We require information from authors about some types of materials, experimental systems and methods used in many studies. Here, indicate whether each material, system or method listed is relevant to your study. If you are not sure if a list item applies to your research, read the appropriate section before selecting a response.

Materials & experimental systems

n/a	Involved in the study	
☐	☒	Antibodies
☐	☒	Eukaryotic cell lines
☒	☐	Palaeontology and archaeology
☐	☒	Animals and other organisms
☒	☐	Clinical data
☒	☐	Dual use research of concern

Methods

n/a	Involved in the study	
☒	☐	ChIP-seq
☒	☐	Flow cytometry
☒	☐	MRI-based neuroimaging

Antibodies

Antibodies used

The following primary antibodies were used in this work: rabbit anti-laminin (1:100, Abcam ab11575), rabbit anti-myc-tag (1:100, Cell Signalling Technology 2272), rabbit anti-fibrinogen (1:100, Abcam ab34269), rat anti-tubulin YL1/2 (1:200, Abcam ab6160), mouse anti-tau (1:500, Sigma-Aldrich MAB3420), chicken anti-MAP2 (1:500, Abcam ab5392), mouse anti-Bassoon (1:250, Enzo SAP7F407), rabbit anti-Homer1 (1:250, Synaptic Systems 160003). The following secondary antibodies were used in this work: goat anti-rabbit IgG Alexa Fluor 594 (Invitrogen A-11037), goat anti-rabbit IgG Alexa Fluor 488 (Invitrogen A-11029), goat anti-rat IgG Alexa Fluor 488 (Invitrogen A-11006). goat anti-chicken IgY DyLight 488 (Invitrogen SA5-10070), goat anti-mouse IgG Alexa Fluor 647 (Invitrogen A-21236), all at a 1:250 dilution (pre-expansion labelling), or 1:500 (post-expansion labelling).

Validation

All primary antibodies used in this study are publicly available. We validated antibodies by confirming expected cellular localisation, in addition to the validation by the manufacturers:
Abcam validation (for ab11575, ab34269, ab6160, ab5392): "IHC and ICC determine whether an antibody recognizes the correct protein based on cellular and subcellular localization. Antibody specificity is confirmed by looking at cells that either do or do not express the target protein within the same tissue. Initially, our scientists will review the available literature to determine the best cell lines and tissues to use for validation. We then check the protein expression by IHC/ICC to see if it has the expected cellular localization (Figure 3). If the localization of the signal is as expected, this antibody will pass and is considered suitable for use in IHC/ICC.".
Cell Signalling Technology validation (for 2272): "All CST™ antibodies that are approved for use in immunofluorescent assays have undergone a rigorous validation process.Validation Steps Include: Cell lines or tissues with known target expression levels are used to verify specificity. Appropriate cell lines and tissues are used to verify subcellular localization. Antibody performance is assessed on appropriate tissues. Cells are subjected to phosphatase treatment to verify phospho-specificity. Target specificity is also verified with the use of known knockout or null cell lines. Cells are subjected to siRNA treatment or over-expression of the target protein to verify target specificity. Activation state specification, target expression, and translocation are examined using ligands or inhibitors to modulate pathway activity. Requirement of threshold signal-to-noise ratio in antibody:isotype comparison and minimum fold-induction for phospho-specific antibodies ensures the greatest possible sensitivity. Fixation and permeabilization conditions are optimized; alternative protocols are recommended if necessary. Stringent testing ensures lot-to-lot consistency."
Sigma-Aldrich validation (for MAB3420): "Immunohistochemistry: Routinely tested on rat brain tissue."
Enzo validation (for SAP7F407): "Purified from hybridoma tissue culture supernatant. Protein G affinity purified."
Synaptic Systems validation (for 160003): "We at Synaptic Systems aim to characterize and validate our products as best as possible to provide antibodies of highest quality and reliability. All our antibodies are produced in-house and therefore we have full control over batch testing and quality control. Specific for Homer 1. Cross-reactivity of the serum to Homer 2 and 3 was removed by pre-adsorption with Homer 2 (aa 1 - 176) and Homer 3 (aa 1 - 177)."

Eukaryotic cell lines

Policy information about cell lines and Sex and Gender in Research

Cell line source(s)

WT U2OS cells were obtained from ATCC. U2OS cells expressing YFP-H2B and mCherry-tubulin were a gift from from Jonne Raaijmakers and René Medema, NKI Amsterdam

Authentication

Cells were authenticated by ATCC, by confirming the STR profile of U2OS.

Mycoplasma contamination

Cells were routinely checked for mycoplasma and discarded if positive.

Commonly misidentified lines
(See ICLAC register)

No commonly misidentified cell lines were used in the study.

Animals and other research organisms

Policy information about studies involving animals; ARRIVE guidelines recommended for reporting animal research, and Sex and Gender in Research

Laboratory animals

10-month-old, TRAP2 heterozygous mice (Jax #030323) were used. Up until the moment of perfusion, mice were group-housed (2–4 per cage) in a temperature- and humidity-controlled room (22 ± 2°C and 60–65% respectively) under a 12 h light/dark cycle (lights on at 7am) with ad libitum access to water and standard laboratory chow [Special Diet Services [SDS], product code CRM(E)].

Wild animals

No wild animals were used in the study.

Reporting on sex	Sex was not considered in sample collection; male mice were used for tissue slices in Fig.4
Field-collected samples	No field collected samples were used in the study.
Ethics oversight	All animal experiments were carried out according to the regulations of Utrecht University and in agreement with Dutch law (Wet op de Dierproeven, 1996) and European regulations (Directive 2010/63/EU).

Note that full information on the approval of the study protocol must also be provided in the manuscript.
